# Resveratrol induced premature senescence and inhibited epithelial-mesenchymal transition of cancer cells via induction of tumor suppressor Rad9

**DOI:** 10.1371/journal.pone.0219317

**Published:** 2019-07-16

**Authors:** Kuan-Yu Chen, Chao-Chung Chen, Yi-Chien Chang, Ming-Chung Chang

**Affiliations:** 1 Hepatobiliary Division, Department of Internal Medicine, Kaohsiung Medical University Hospital, Kaohsiung Medical University, Kaohsiung, Taiwan; 2 Department of Biotechnology, College of Medical and Health Care, Hung Kuang University, Taichung, Taiwan; 3 Department of Surgery, National Cheng Kung University Medical College and Hospital, Tainan, Taiwan; 4 Division of Thoracic Surgery, Tainan Municipal Hospital, Show Chwan Health Care System, Tainan, Taiwan; 5 Department of Nutrition, College of Medical and Health Care, Hung Kuang University, Taichung, Taiwan; Seoul National University College of Pharmacy, REPUBLIC OF KOREA

## Abstract

Resveratrol (RSV) has been reported to influence many biological processes, including the stimulation of cellular senescence and inhibition of epithelial-mesenchymal transition (EMT). In this research, we explored the mechanisms of RSV on EMT and cellular senescence through the expression of a DNA damage response (DDR) protein, Rad9, in breast and lung cancer cell lines. Upon treating breast and lung cancer cell lines with RSV at the concentrations of 10–50 μM, Rad9 expression was increased at both transcriptional and translational levels. The results indicated that RSV-induced Rad9 expression, mediated by DNA damage and ROS, can significantly suppress proliferation by activating cellular senescence, and diminishing the expression of EMT markers with concomitant downregulation of Slug in breast and lung cancer cell lines. By using a siRNA approach, RSV was shown to mediate cellular senescence and EMT through a Rad9-dependent mechanism. The treatment with RSV can inhibit the proliferation, EMT, and increase cellular senescence of breast and lung cancer cell lines by activating Rad9. Our results suggest that the breast and lung tumor suppressive activities of RSV are, at least in part, mediated by the upregulation of Rad9.

## Introduction

Senescence is an irreversible form of cell-cycle arrest that can be triggered by various forms of intrinsic and extrinsic stresses. Several studies have demonstrated that the induction of cellular senescence could occur *in vivo* and the bypass of cellular senescence is an important step in tumorigenesis [1]. Senescence is well regarded as a crucial tumor suppressive mechanism and the induction of senescence is a promising alternative strategy for the treatment and prevention of cancer [2]. Another cellular mechanism, the epithelial-mesenchymal transition (EMT) is an essential developmental process by which cells with epithelial origin can lose their epithelial characteristics and acquire a mesenchymal phenotype with increased cell motility and invasiveness. This EMT mechanism also plays an important role in tumor invasion and metastasis [3]. The upregulation of EMT transcription factors such as Snail, Twist, and Zeb family proteins is associated with increased malignant phenotypes in variety of human cancers. A growing body of experimental evidence has shown that EMT and senescence are cross-interacting during tumor progression. For example, a number of key senescence-associated factors, such as p53, p21, and RB, have been found to affect EMT [4–6]. Whereas, several distinct EMT transcription factors, such as Snail, Twist, and Zeb1 can also concomitantly suppress senescence [7–9].

Previous studies have shown that a DNA damage response (DDR), triggered by uncapped telomeres or non-telomeric DNA damage, is the most prominent initiator of senescence. The DDR is characterized by the activation of sensor kinases that can stimulate the formation of DNA damage foci containing activated H2AX (gamma-H2AX) and ultimately the induction of cell cycle arrest through activation of the p53- p21 pathway[10]. In a recent study, we have reported that Rad9 protein, one of the key players in the DDR, functions as a tumor suppressor by inducing p21-dependent senescence in breast and lung cancers [11]. The reduction of Rad9 expression was found in most breast and lung cancer specimens, especially in patients with invasive breast and lung cancers. This reduction in Rad9 expression was also found in the highly invasive breast cancer and lung cancer cell lines, MDA-MB 231 and H1299, respectively. In addition, ectopic Rad9 expression in MDA-MB 231 cells and H1299 cells resulted in induced senescence by the upregulation of p21 and the simultaneous attenuation of cellular migration and invasion abilities with concomitant downregulation of Slug and thereby, suppressed the tumorigenicity *in vitro* and in a xenograft mouse model [11]. Although Rad9 is a potential tumor suppressor in breast and lung cancers, and can selectively regulate genes that contribute to DDR or EMT, such as p21 [12], NEIL1 [13], and Slug [11], other Rad9-targeting genes and factors that may down- or up-regulate the transcription of Rad9 are not yet to be identified [14].

Resveratrol (trans-3,4’,5-trihydroxystibene; RSV), is a polyphenolic phytoalexin that is present in many plants and is abundantly in grapes and red wine [15]. RSV has been proposed as an ideal chemopreventive and chemotherapeutic agent due to its relatively low toxicity to normal cells and its capacity to inhibit the proliferation of a wide variety of human tumor cells *in vitro* and in xenograft models [16]. Furthermore, RSV is safety for its antitumor mechanism in healthy volunteer’s trail [16]. In addition to the inhibition of tumor cell proliferation, the anti-tumor properties of RSV also included its ability to induce apoptosis, autophagic-related cell death, and senescence and to inhibit EMT, invasion, angiogenesis, and metastasis [17]. Although the positive biological effects and anti-tumor properties of RSV are very well demonstrated, the precise mechanisms involved in RSV-mediated tumor-suppressing activities have only been understood partially.

In the present study, a novel mechanism of breast and lung cancers prevention by RSV involving Rad9 DDR protein was investigated. A significant up-regulation in the expression of Rad9 was determined at both transcriptional and translational levels upon the treatment of breast and lung cancer cell lines with RSV at the concentrations of 10 to 50 μM. The results of this study provided experimental evidence that RSV exerts its anticancer effects, at least in part, via DDR activation- and the production of reactive oxygen species (ROS) mediated upregulation of Rad9 in breast and lung cancer cell lines.

## Materials and methods

### Reagents

Resveratrol (Trans-3, 4 ', 5-trihydroxystilnene), nucleosides (NS), and N-acetylcysteine (NAC) were purchased from Sigma-Aldrich (St. Louis, MO, United Stated). Phorbol-12-Myristate-13-Acetate (TPA) was purchased from Cell Signaling (Danvers, MA, United States). Dulbecco’s modified Eagle’s medium (DMEM) and other culture media were obtained from Invitrogen (Carlsbad, CA, United States). A senescence associated β-galactosidase (SA-β-gal) staining kit was purchased from Cell Signaling (Danvers, MA, United States). The antibodies used for the Western blot including p53, p21, Rad9, Slug, E-cadherin, and vimentin were purchased from Genetex (San Antonio, TX, United States). Antibodies such as γ-catenin, N-cadherin, and elongation factor 1 alpha (EF1A) were purchased from Abcam (Cambridge, UK). The antibodies including β-Actin, α-tubulin, and GAPDH were purchased from Proteintech Group Inc (USA). The HRP-conjugated goat anti-mouse and anti-rabbit secondary antibodies were purchased from Abcam (Cambridge, UK).

### Cell culture

The MCF-7 and A549 cell lines were cultured in complete medium consisting of Dulbecco’s Modified Eagle’s medium (DMEM, Gibco, Grand Island, NY, USA) and 10% fetal bovine serum (FBS, Invitrogen, Carlsbad, CA, USA). All cells were incubated in 10-cm tissue culture dishes at 37°C and 5% CO_2_; and were sub-cultured every 3–4 days. The MCF-7 and A549 cell lines were purchased from Bioresource Collection and Research Center (BCRC) in Hsinchu, Taiwan.

### siRNA knockdown studies and real time PCR

The transfection of siRNA was performed using Lipofectamine RNAiMAX (Invitrogen, Carlsbad, CA, United States) according to the manufacturer’s instructions. The siRNA sequences included the followings (TOOLS, Taiwan): An siRNA targeting the wild-type firefly Luciferase gene was used as a scrambled control, 5'-CUCUAUCAUU GAUAGAGUU-3′; and a specific siRNA sequence targeting Rad9 (GenBank^™^ accession number U53174) corresponded to the start codon (coding region 232–252: 5′-GUCUUUCCUG UCUGUCUUC-3′)[11, 18–20]. The total RNA was isolated with Mini RNA Isolation Kit (Zymo Research, Orange, CA, United States) and reverse transcribed using Superscript III RT (Invitrogen, Carlsbad, CA, United States) according to the manufacturer’s instructions. Equal amounts of cDNA were subjected to realtime PCR using SYBR-Green PCR master mix (Applied Biosystems, CA, United States) in an ABI 7900 real-time PCR system. The following primer pairs were used: Rad9, 5′-CTCACTGGCG ATGCTGGAGA-3′ (forward) and 5′-TTGCAATGCA GCTGGACCAC-3′ (reverse); β-Actin, 5′-GAGCTAGG AG CTGCCTGAC-3′ (forward) and 5′-AGCACTGTGT TGGCGTACAG-3′ (reverse). All experiments were repeated at least three times.

### Clonogenic survival and senescence assays

Clonogenic assays were performed to determine the effects of RSV treatment on the colony-forming ability of MCF-7 and A549 cell lines. A total of 500 cells/well were plated into 60-mm culture dishes in triplicates. After a 12-day incubation at 37°C and 5% CO_2_, cells were fixed with formaldehyde and stained with 2% crystal violet. The number of colonies was then counted, and the surviving fraction of treated cells was normalized to the surviving fraction of the corresponding controls. All experiments were repeated at least three times. Senescent cells were examined by SA-β-Gal staining. The general staining procedures were performed as the followings in brief: Cells were fixed in 0.2% (wt/vol) glutaraldehyde and then stained with the staining solution 5 mM K_3_Fe (CN)_6_, 5 mM K_4_Fe (CN)_6_; 30 mM sodium phosphate buffer; 150mM NaCl; 2mM MgCl_2_; and 1 mg/ml of X-Gal at pH 6.0 at 37°C for 16–24 h. At least 500 cells were counted for each sample.

### Scratch wound healing assay

After the cells have become confluent, a wound field was induced using a pipette tip. The cells were then placed in a fresh serum-free medium and divided into 4 groups including: control group (MCF-7 and A549), RSV-treated group (MCF-7 and A549), RSV-treated with siRNA control group (MCF-7 and A549), and RSV-treated with siRad9 group (MCF-7 and A549). The wounds were examined under a Nikon Eclipse Ti-U fluorescent microscope (Nikon Instruments Inc., Japan). All experiments were repeated at least three times.

### Cell invasion assay

The invasion of cancer cell lines was performed in the transwell chambers. Matrigel was used to coat the Millicell culture plate filter inserts (pore size, 8.0 μm). The cells were suspended (100 μL containing 2×10^4^ cells) in DMEM containing 1% FBS and then added to the upper chambers. Simultaneously, 500 ml of DMEM containing 20% FBS was placed in the lower chambers. The cells were allowed to migrate for 48 h at 37°C. The non-migrated cells were removed from the upper surface by scraping with a wet cotton swab. After rinsing with PBS, the filter was fixed and stained with crystal violet. The numbers of cells in five random fields were counted with the use of a microscope under 100 times magnification. The mean values of the data were obtained from the chambers of three separate chambers. All experiments were repeated at least three times.

### Immunofluorescence staining of gamma-H2AX

Cells were grown on cover slips and treated with RSV or 0.01% dimethyl sulfoxide (DMSO). After treatment, the cells were fixed with 3.7% formaldehyde in PBS buffer, and permeabilized with 0.2% Triton X-100, and blocked in PBS containing 5% bovine serum albumin (BSA). Rabbit anti- gamma -H2AX antibody (Millipore, Billerica, MA, United States) was then added at a dilution of 1:200 in 5% Fetal bovine serum (FBS) in PBS and incubated overnight at 4 °C. The following day of the incubation, cells were washed and incubated for one hour at 37°C in the dark with anti-rabbit Alexa 594 (Invitrogen, Carlsbad, CA, United States) at a dilution of 1:500 with 5% FBS in PBS for 60 min. Cells were then incubated, in the dark with 4-6-diamidino-2-phenylindole (DAPI), for additional 5 min onto microscope coverslips and examined under a fluorescent confocal microscope (Zeiss LSM 700 confocal laser scanning microscope). At least 100 cells were counted for each sample.

### Measurement of ROS

MCF-7 and A549 cell lines treated with NAC or NAC and RSV, the cells were incubated with PBS and 10 μM oxidation sensitive fluorescent probe (DCFH-DA) for 20 min at 37°C in the 5% CO_2_ incubator. Cells were then examined under the fluorescent microscope. All experiments were repeated at least three times.

### Western blotting

The control samples consisted with MCF-7 and A549 cells treated with different doses of RSV or DMSO. Total cell proteins were prepared at 24 h post treatment using cell lysis buffer (Cell Signaling Technology, Danvers, USA) supplemented with a cocktail of proteinase inhibitors (Sigma-Adrich). The Western blot analysis was performed as described in a previously published study by our research group [21]. Briefly, total protein was resolved by SDS-PAGE and transferred onto a polyvinylidene difluoride membranes (Millipore, USA) and blocked with 5% nonfat dry milk. The membrane was incubated with a primary antibody, followed by a secondary antibody coupled to HRP. The target bands were developed with ECL substrate (WBKLS0500; Millipore, USA). All experiments were repeated at least three times. The band intensities in the immunoblotting were determined by ImageJ software.

### Statistical analyses

All experiments were repeated independently for at least three times. Paired comparisons were performed using Student’s t-test, and values of p < 0.05 were considered statistically significant. Data are expressed as mean ± standard deviation (SD). All analyses were performed with the GraphPad Prism program by GraphPad Software, Inc. (San Diego, CA, United States).

## Results

### RSV induced premature senescence in breast and lung cancer cells

**Fig 1:**
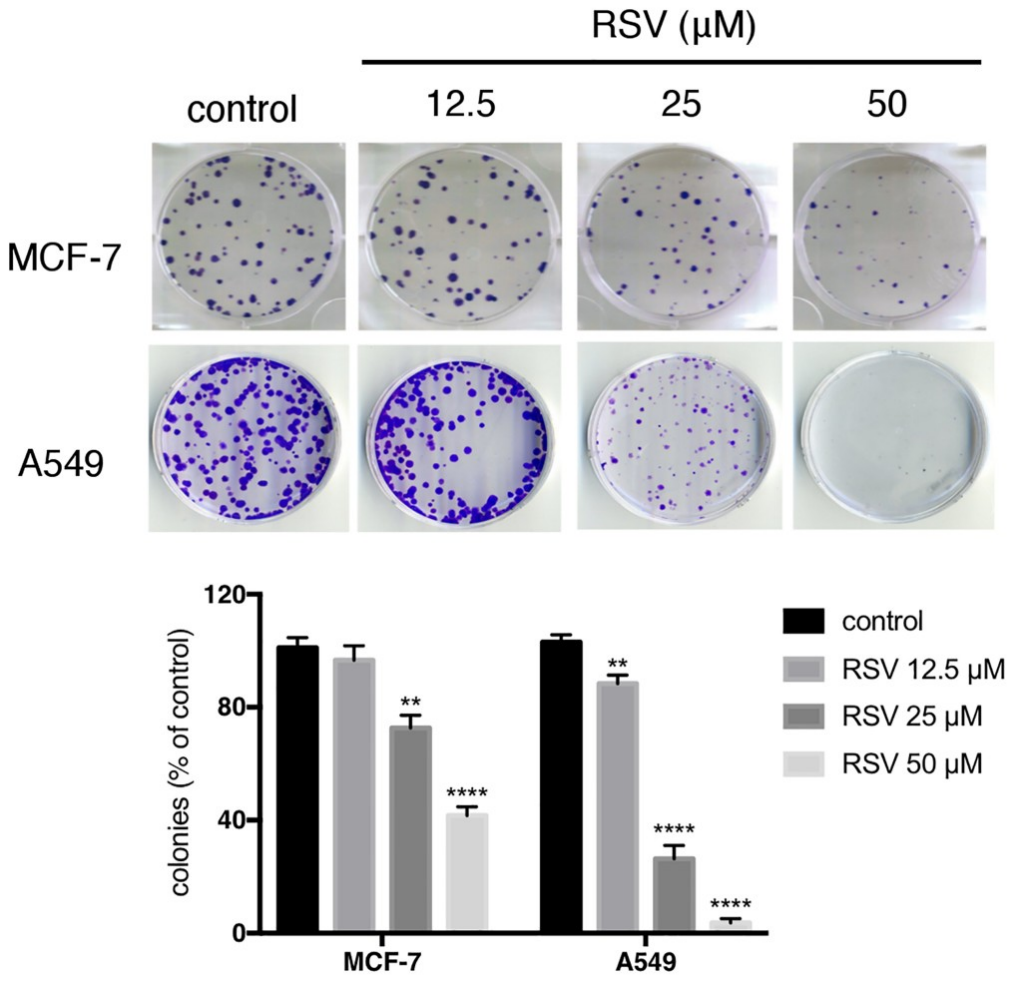
The inhibition of the growth of MCF-7 and A549 cell lines in a dose-dependent manner by RSV. Clonogenic survival assays of MCF-7 and A549 cells (500 cells/well) were seeded in a 6-well dish and treated with the indicated concentrations of resveratrol (RSV). After 2 weeks of incubation, the number of MCF-7 and A549 cell-derived colonies decreases with increased RSV dose. The MCF-7 or A549 cell clones (500 cells/well) were seeded in a 6-well dish. After 12 days of incubation, the cells were stained with crystal violet and counted by phase-contrast microscopy. Bars represent means ± standard deviation (s.d.), and asterisks denote a statistically significant difference. Data are representative of at least three independent experiments performed in triplicates. **, **** P≤0.05 based on Student’s t-test.

To assess the potential anti-tumor activity of RSV, this study was started with treating the MCF-7 breast cancer cell line and A549 lung cancer cell line with different concentrations of RSV for 24 h. Although a concentration of < 10 μM of RSV did not affect the proliferation of MCF-7 and A549 cells, RSV at a concentration of > 100 μM exhibited the induction of rapid cell death in both cell lines. This result suggests that both the MCF-7 and A549 cells undergo apoptosis following exposure to 100 μM RSV, which is consistent with previous studies demonstrated that higher doses (> 100 μM) of RSV can inhibited the growth of breast cancer cells by inducing apoptosis through the activation of cell death signaling [22]. In addition, our results are also consistent with previous study that demonstrated RSV treatment at a relatively low dose can suppress anchorage-dependent colony formation ability of A549 cells [23]. In our study, RSV treatment (12.5–50 μM) of MCF-7 and A549 cells can significantly suppress colony formation ability of both cell lines in a dose-dependent manner (Fig 1).

**Fig 2:**
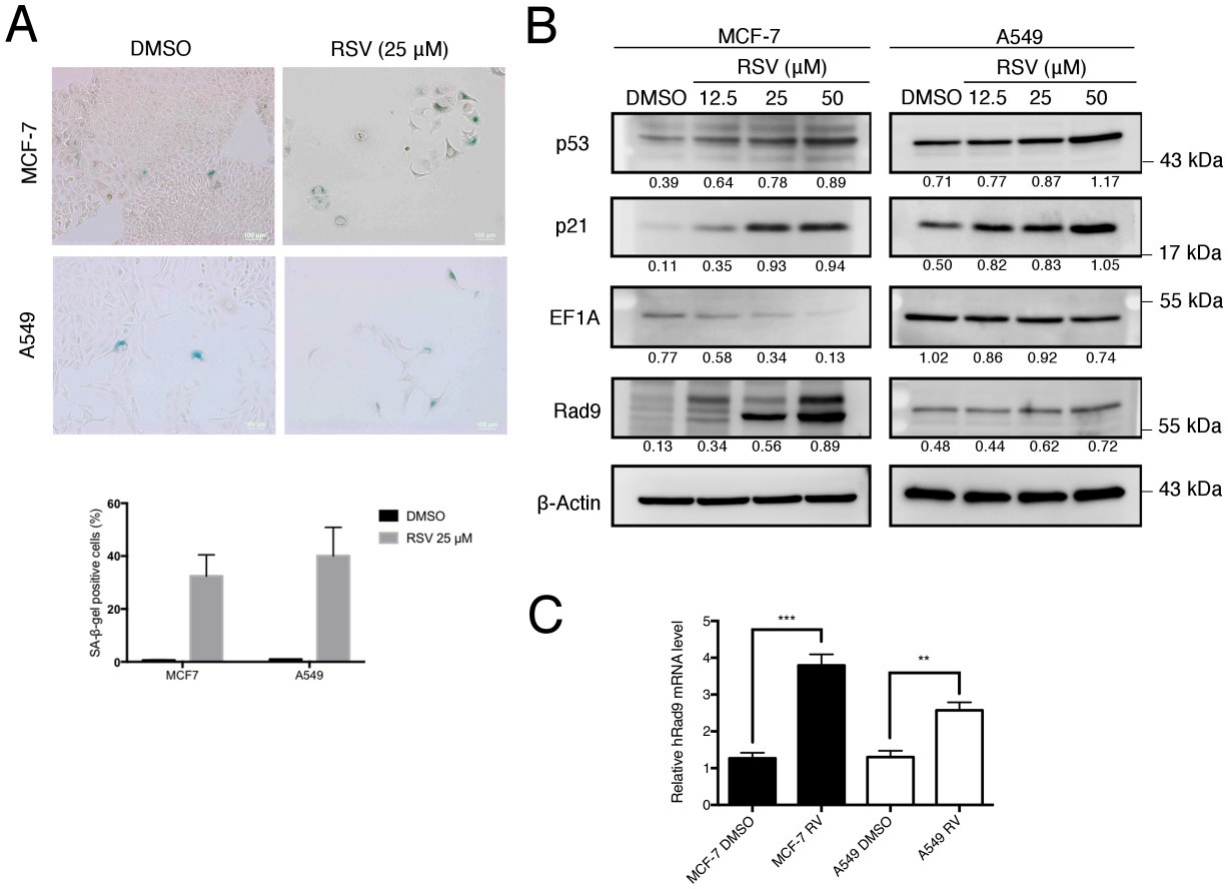
Rad9 involved RSV induction of premature senescence in MCF-7 and A549 cell lines. (A) SA-β-gal assays were performed 7 days after treated with 25 μM of RSV in MCF-7 and A549 cell lines. Scale bar, 100 μm, representative of three independent experiments. (B) Western blotting was performed to determine the expression of p53, p21, Rad9, and EF1A in RSV treated MCF-7 and A549 cells. β-Actin was used as a loading control. (C) Real-time PCR was performed to determine Rad9 gene expression in RSV treated cells. Data are means ±s.d., **, **** P≤0.05 (control vs. each treatment), representative of three independent experiments. Signals on the western blots were analyzed by ImageJ, normalized with that of β-Actin.

It has been reported that low dose RSV can induce premature senescence through the p53- and p21-associated pathway in A549 cells [23]. Thus, the ability of low dose RSV for the induction of premature senescence in MCF-7 cells was investigated. The numbers of SA-β-gal positive senescence cells are also markedly increased in RSV-treated-cells in comparison to the control MCF-7 and A549 cells (Fig 2A). This result demonstrated that 25 μM of RSV treatment can induce premature senescence in MCF-7 and A549 cells. In addition, Western blotting demonstrated that p53 and p21 proteins were significantly increased in RSV-treated MCF-7 and A549 cells in a dose-dependent manner when compared to their respective controls (Fig 2B).

### RSV treatment increased Rad9 protein levels

Previous reports have shown that DDR is a crucial mediator of cellular senescence [10]. Since we have previously shown that a DDR protein, Rad9, can elicit tumor suppression by inducing p21-dependent senescence in breast and lung cancers [11], the role of Rad9 was investigated in RSV treatment induced premature senescence by evaluating the mRNA levels of Rad9 using quantitative RT-PCR. The results indicated that compared to mock-treated cells, Rad9 mRNA levels were increased in cells treated with 25 μM of RSV (Fig 2C). Furthermore, the Western blotting revealed that, in both cell lines in a dose-dependent manner, 12.5–50 μM of RSV treatment can significantly up-regulate the expression levels of Rad9, while significantly downregulate the expression levels of EF1A, a potential biomarker for premature senescence [24] (Fig 2B). These results suggested that 12.5–50 μM of RSV treatment can inhibit clonogenic growth, and induce premature senescence by upregulating p53 and 21, and downregulating EF1A in both breast and lung cancer cells. RSV treatment (12.5–50 μM) can also significantly increase Rad9 transcript and protein levels, suggesting that besides p53, p21 and EF1A, Rad9 upregulation is also involved in RSV-induced premature senescence in both cell lines.

### RSV induced Rad9 upregulation attenuated by nucleosides and n-acetylcysteine (NAC)

**Fig 3:**
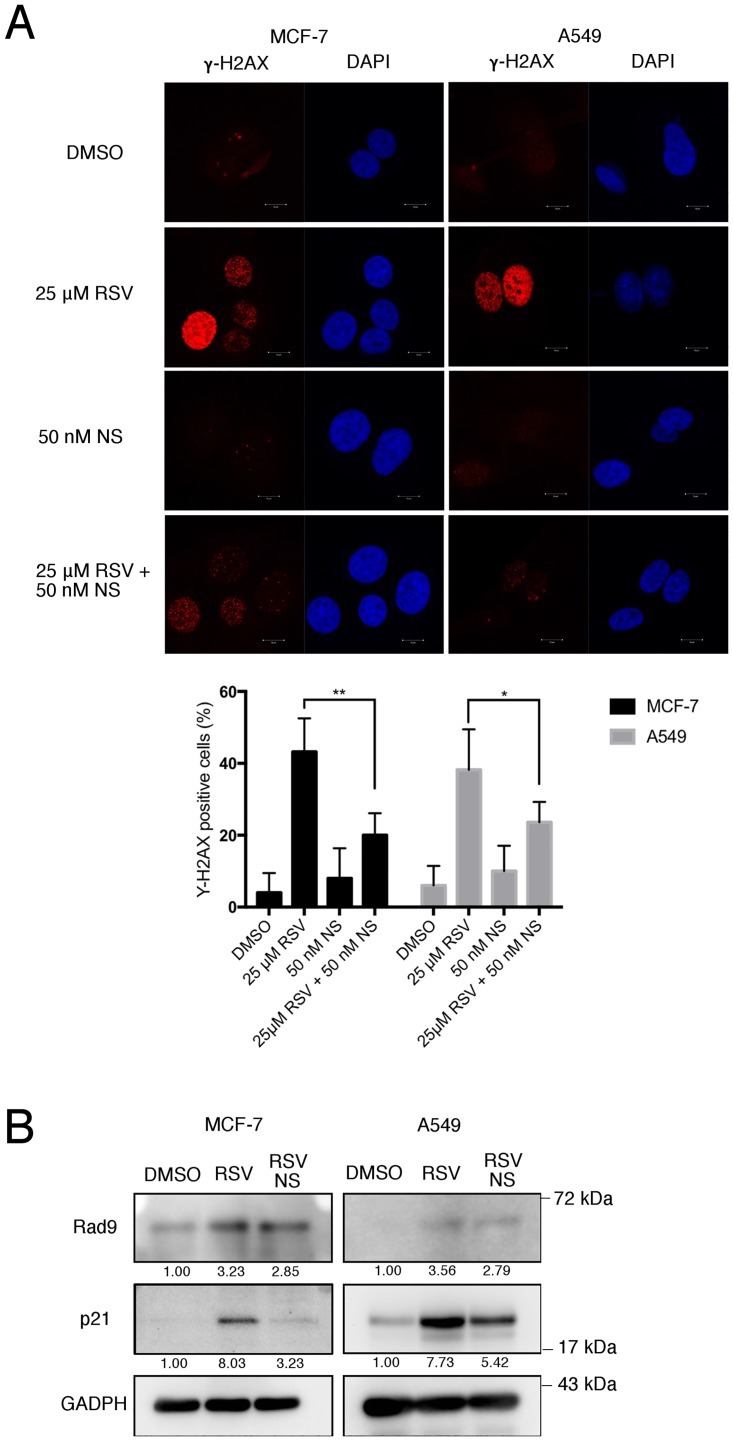
RSV induced Rad9 upregulation attenuated by nucleosides. (A) Immunofluorescence of gamma-H2AX in RSV treated MCF-7 and A549 cell lines. Cells were treated with RSV (25 μM) or in combination with nucleosides (NS, 50 nM) for 24 h; stained with gamma-H2AX(Ser139) antibody (red) and counterstained with DAPI (blue). Scale bar, 10 μm, representative of three independent experiments. (B) Western blotting was performed to determine the expression of Rad9 and p21 in MCF-7 and A549 cells treated with RSV (25 μM) or in combination with NS (50 nM) for 24 h. Signals on the immunoblots were analyzed by ImageJ, normalized with that of GAPDH.

Previous studies have demonstrated that the anticancer effects of RSV were linked to its ability to modulate proteins involved in DDR signaling [23, 25, 26]. In line with these studies, a remarkable increase in the amount of gamma-H2AX, a hallmark of DNA damage response, in RSV-treated-MCF-7 cells and A549 cells were observed (Fig 3A). The results indicated that DNA damage is involved in the RSV-elicited induction of premature senescence in both cell lines.

It has been reported that RSV can act as an inhibitor of ribonucleotide reductase and DNA damage induced by RSV can be attenuated by exogenous nucleosides [27]. Since it was demonstrated in this study that RSV induced senescence is mediated by DNA damage and upregulation of Rad9 expression in both examined cell lines, we speculated that the induction of DNA damage by RSV may be responsible for the upregulation of Rad9 expression. To test this hypothesis, we examined if RSV-induced Rad9 upregulation can be affected by nucleosides. The results demonstrated that RSV-induced upregulation of Rad9 expression was significantly attenuated by nucleosides (Fig 3B). Similar response to the reduction in DNA damage and senescence, p21 upregulation was also shown to be attenuated by nucleosides (Fig 3B). This result suggested that DNA damage is involved in the RSV-elicited upregulation of Rad9, resulting in the induction of premature senescence in both breast and lung cancer cells.

**Fig 4:**
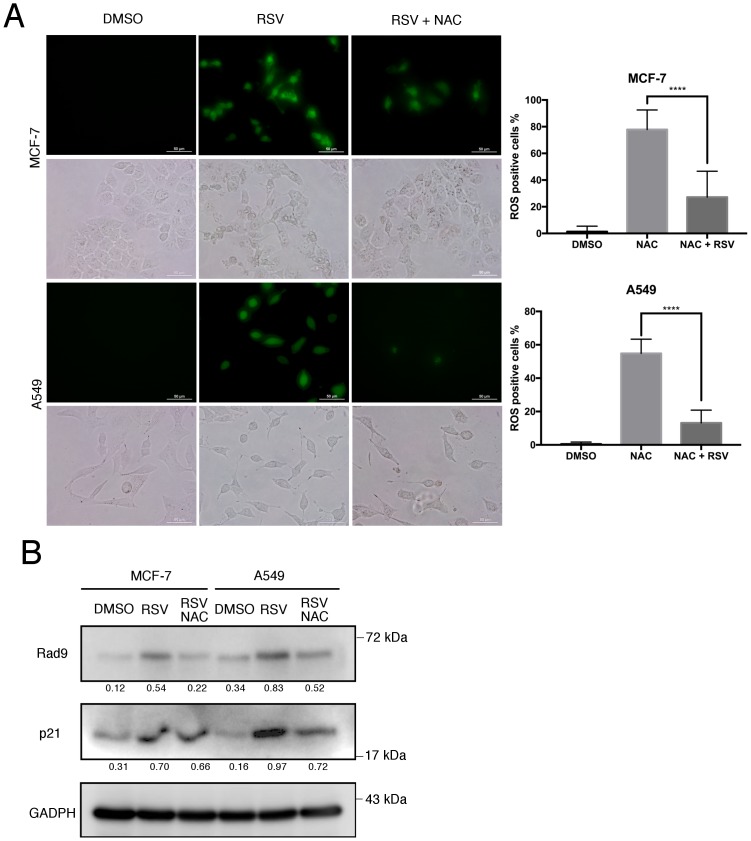
RSV induced Rad9 upregulation attenuated by NAC. (A) Green fluorescence of dichlorodihydrofluorescein (DCF) probe was used the quantitation of ROS in MCF-7 and A549 cells treated with RSV (25 μM) with or without NAC (10 mM) for 24 h. Scale bar, 50 μm, representative of three independent experiments. Data are means ±s.d., **** P≤0.05. (B) MCF-7 and A549 cells were exposed to RSV with or without NAC (10 mM) for 24 h. The expression of Rad9 and p21 were determined by immunoblotting. Signals on the immunoblots were analyzed by ImageJ, normalized with that of GAPDH.

Previous studies have reported that in addition to DNA damage, oxidative stress was also shown to be a key mediator of RSV-induced senescence in various human cell lines (including A549 cells) [23, 26, 27]. Therefore, the levels reactive oxygen species (ROS) were measured in both of the RSV exposed cell lines. The results indicated that ROS levels were significantly elevated in both RSV-treated MCF-7 and A549 cells, when compared with their respective controls (Fig 4A). To further examine if RSV-induced Rad9 upregulation can be affected by ROS levels, a ROS inhibitor, N-acetycysteine (NAC) was introduced to the exposed cells. The results exhibited that RSV-induced upregulations of Rad9 and p21 expressions were significantly attenuated by NAC (Fig 4B), suggesting that ROS has a role in the RSV-elicited upregulation of Rad9. The combination of these results revealed that RSV induced cellular senescence is achieved by the upregulation of Rad9, which is mediated by the activation of DDR and elevation of ROS levels. The results suggest that RSV could induce DNA damages and elevate ROS levels, which in turn upregulate Rad9, and result in the subsequent induction of p21, resulting in the induction of cellular senescence.

### Inhibition of RSV-suppressed clonogenic growth and -induced premature senescence by silencing Rad9, and the blocking of RSV-inhibited cell mobility and mesenchymal-epithelial transition (EMT)

**Fig 5:**
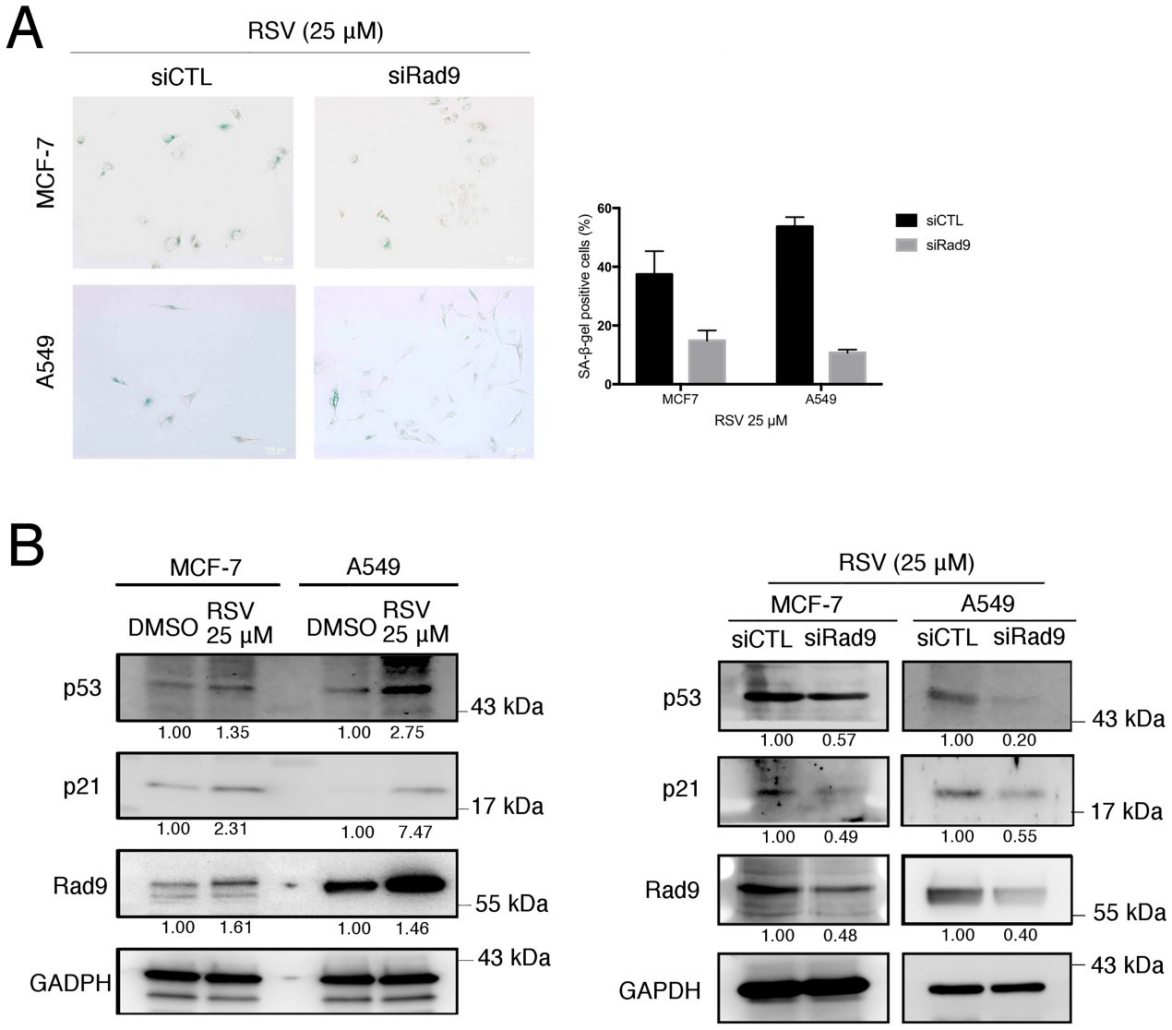
RSV induced premature senescence in a Rad9-dependent manner. (A) SA-β-gal staining was reduced in RSV treated siRad9/MCF-7 and siRad9/A549 cells. Scale bar, 100 μm, representative of three independent experiments. (B) Western blotting was performed to determine the expression of p53, p21, and Rad9 in RSV or with siRad9 treated in MCF-7 and A549 cells. GAPDH were used as loading controls. Signals on the immunoblots were analyzed by ImageJ, normalized with that of GAPDH.

To further demonstrated that Rad9 is involved in RSV-suppressed clonogenic growth and induced premature senescence, Rad9 expression was silenced in MCF-7 and A549 cells and their cellular behaviors examined. The results indicated that SA-β-gal positive senescence cells were significantly decreased in RSV-treated MCF-7 and A549 cells, followed by siRNA directed knockout of Rad9 (Fig 5A). In addition, Western blotting also revealed that the knockdown of endogenous Rad9 in MCF-7 cells and A549 cells significantly reduced RSV-mediated upregulations of p53 and p21(Fig 5B). These results indicated that RSV treatment suppressed clonogenic growth as well as induced premature senescence by upregulating p53 and p21 in MCF-7 cells and A549 cells are least mediated in part by the upregulation of Rad9.

**Fig 6:**
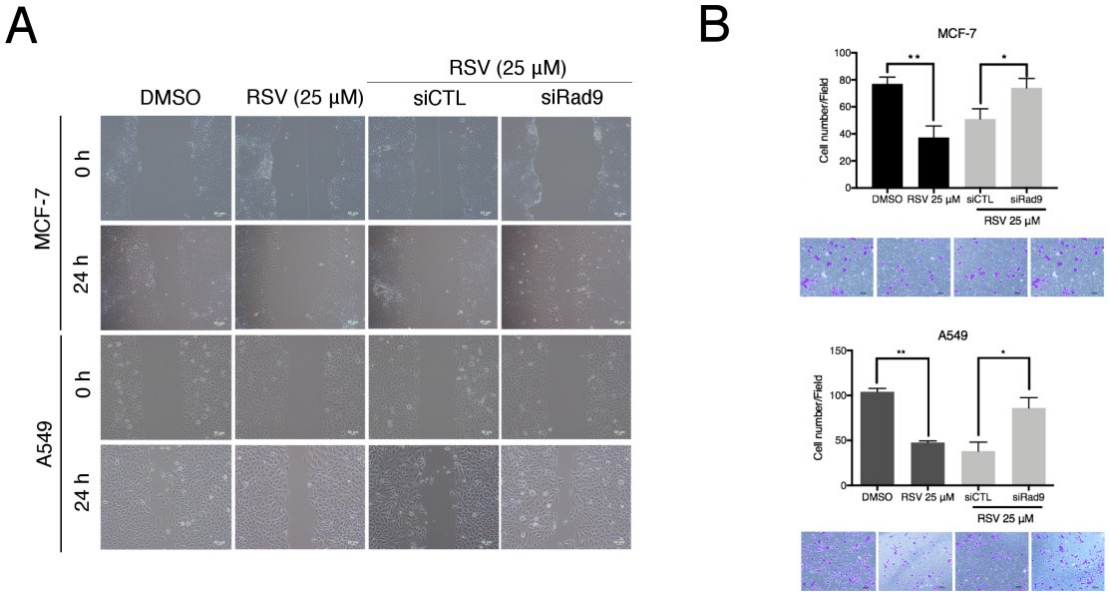
RSV inhibited cell mobility in a Rad9-dependent manner. (A) Scratch wound healing assay was performed to examine the effects of RSV treatment and Rad9 gene silencing on MCF-7 and A549 cells. Scale bar, 100 μm. (B) The effects of RSV treatment and Rad9 gene silencing on MCF-7 and A549 cells invasion. Cells that had invaded to the bottom of the membrane were counted manually after staining with crystal violet. Scale bar, 100 μm. **, *** P≤0.05 based on Student’s t-test.

**Fig 7:**
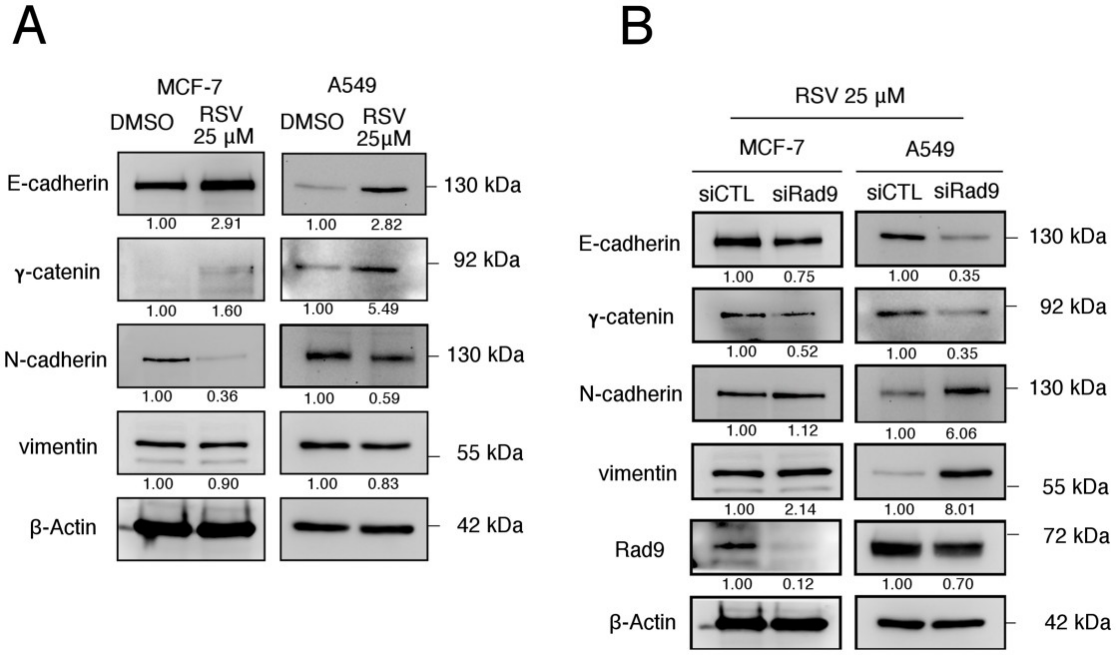
RSV inhibited EMT in a Rad9-dependent manner. (A) Western blotting was used to examine the effect of RSV treatment on EMT related protein expression. (B) Immunoblotting analysis was carried out to determine the effects of RSV treatment and Rad9 gene silencing on EMT related protein expression. β-Actin was used as a loading control. Signals on the western blots were analyzed by ImageJ, normalized with that of β-Actin.

Since our previously published study has demonstrated that Rad9 functions as a tumor suppressor by attenuating cellular migration and invasion, and simultaneously inhibiting the EMT in breast and lung cancer cell lines, the involvement of Rad9 in RSV-inhibited cell mobility and induced mesenchymal-epithelial transition (MET) in MCF-7 and A549 cells is further examined. The results revealed that RSV-treated cells exhibited significantly decreased migration and invasion abilities. This RSV-mediated inhibition of migration and invasion abilities in MCF-7 and A549 cells was shown to be reduced by the knockdown of endogenous Rad9 (Fig 6). Furthermore, an increase in epithelial markers, E-cadherin and γ-catenin, and a decrease in mesenchymal markers, N-cadherin and vimentin, in RSV-treated cells were observed. The opposite trends in the expressions of these proteins were observed in RSV-treated cells with siRNA directed against Rad9 when compared to nonspecific siRNA knockdown (Fig 7). These results indicated that the upregulation of Rad9 is important for RSV-suppressed cellular migration and invasion, and simultaneously inhibited EMT in breast and lung cancer cell lines.

### Inhibition of Slug expression via upregulation of Rad9 by RSV in A549 and MCF-7 cells

**Fig 8:**
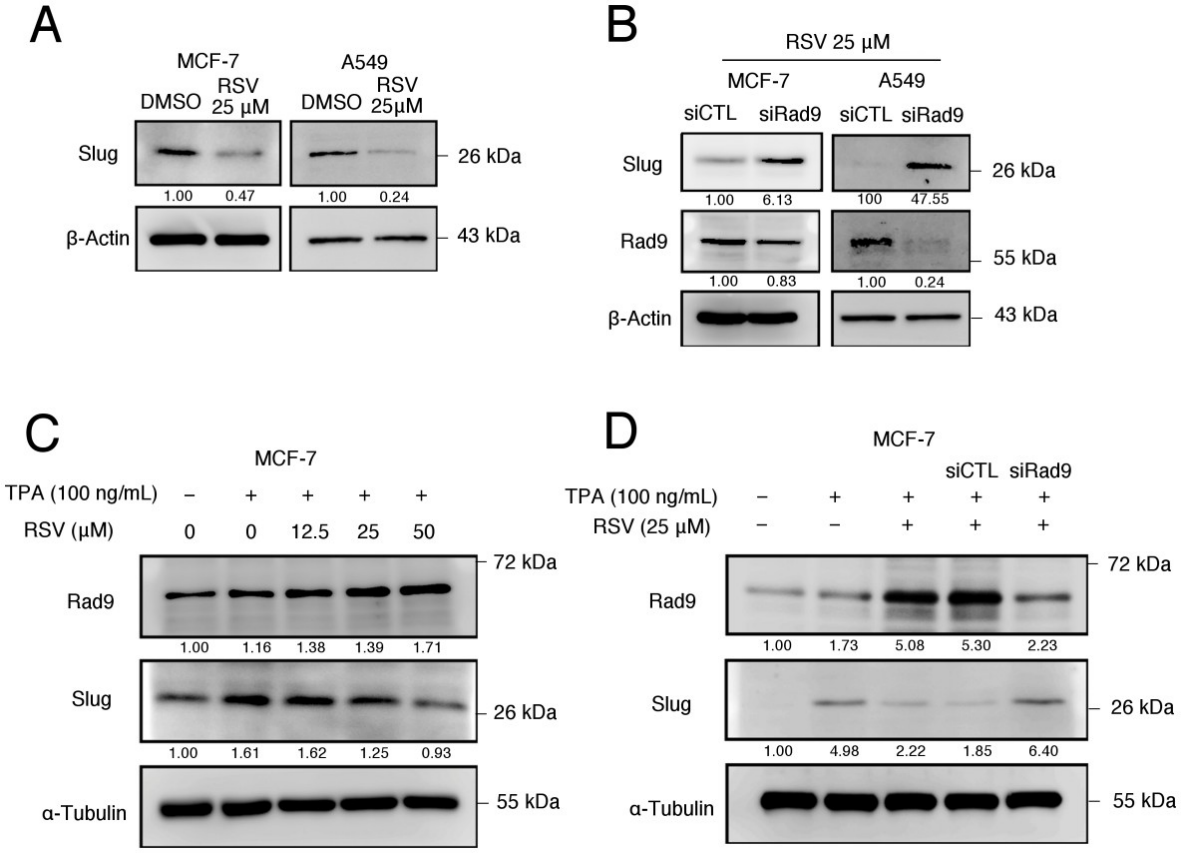
RSV inhibited Slug expression in a Rad9-dependent manner. (A) Western blotting was used to examine the effects of RSV treatment on Slug and Rad9 protein expression. (B) Immunoblotting analysis was performed to determine the effects of RSV treatment and Rad9 gene silencing on Slug protein expression. (C) Western blotting was used to examine the effect of phorbol-12-myristate-13-acetate (TPA) treatment with or without RSV on Rad9 and Slug protein expression. (D) Immunoblotting analysis was performed to determine the effects of TPA treatment with or without RSV and Rad9 gene silencing on Rad9 and Slug protein expression. β-Actin and α-tubulin were used as a loading control. Signals on the western blots were analyzed by ImageJ, normalized with that of β-Actin or α-Tubulin.

Our previously published study has demonstrated that Rad9 plays an important role in the inhibition of EMT. This inhibition is a result of Rad9 binding directly to the promoter region of Slug transcription factor, one of important EMT inducers, and repressed its transcriptional activity [11]. The inhibition of EMT by RSV in breast and lung cancer cell lines via the upregulation of Rad9 with concomitant downregulation of Slug is thus further investigated. The Western blotting indicated that treatment with RSV can significantly decreased the expression of Slug in both cell lines, with a more pronounced effect in A549 cells (Fig 8A). Furthermore, the RSV-mediated downregulation of Slug is rescued in knockdowns of endogenous Rad9 in MCF-7 and A549 cells (Fig 8B).

### Inhibition of TPA-elicited Slug induction via upregulation of Rad9 in MCF-7 cells by RSV

A recent study reported that MCF-7 cells treated with phorbol ester 12-O-tetradecanoylphorbol-13-acetate (TPA) resulted in specifically induced upregulation of Slug [28]. Therefore, we investigated if TPA-elicited Slug induction may also be regulated by Rad9 in MCF-7 cells. As expected, the TPA treatment induced the expression of Slug in MCF-7 cells. In the presence of RSV, the TPA-elicited induction of Slug was, however, significantly reduced (Fig 8C). The knockdown of endogenous Rad9 in MCF-7 cells was shown with rescued RSV-mediated downregulation of Slug, when compared with knockdown of nonspecific siRNA (Fig 8D). Collectively, our data demonstrated that the inhibition of EMT in breast and lung cancer cell lines by RSV is largely mediated via the upregulation of Rad9 with the concomitant downregulation of Slug.

## Discussion

In the present study, it was demonstrated that 12.5–50 μM of RSV can decrease the proliferation of breast cancer and lung cancer cells in a time and dose dependent manner that is associated with the induction of premature senescence. Consistent with a previous study that demonstrated low doses RSV induced senescence in lung cancer cells [23], we demonstrated that similar concentrations of RSV could induce premature senescence in breast and lung cancer cells in this study. More importantly, we demonstrated for the first time that RSV induced senescence is associated with a concomitant increase in Rad9 at both transcriptional and translational levels. A number of evidences were presented for the upregulation of Rad9 by RSV elicited anti-tumorigenic effects of breast and lung cancer cell lines through a multitude of signaling pathways. These pathways included reduced cell growth, induced premature senescence, suppressed cellular migration and invasion, inhibited EMT, and upregulated transcription of Slug.

It is well documented that senescence-inducing signals are established and maintained by either the p53-p21 and/or p16/pRb pathway. Both of the cyclin dependent kinase inhibitors p21 and p16 are likely to cooperate to maintain pRb in a hypo-phosphorylated form during cellular senescence [1]. Intriguingly, because both MCF-7 and A549 cells lack the p16 gene, the p53-p21 pathway is postulated to play a pivotal role in RSV induced cellular senescence. In this study, we demonstrated that RSV induced premature senescence is associated with a significant increase in p53 and p21 protein levels in MCF-7 and A549 cells, suggesting that activation of the p53-p21 pathway may play an important role in modulating RSV-induced senescence in both cell lines. It has been reported that ROS can damage DNA directly and thus induce DDR and senescence. Conversely, the activation of major downstream effectors of the DDR checkpoints can also induce ROS production [29, 30]. Reports have also indicated that the senescence induction ability and anticancer properties of RSV are mostly related to DDR-mediated cell arrest and oxidative stress [23, 25, 26]. A recent study has also reported that RSV can sequentially induce replication stresses (DDR-mediated S phase arrest) and oxidative stresses in U2OS, A549, and normal human fibroblasts (NHFs) cells. This study further demonstrated that while the induction of ROS by RSV occurred after and was independent of S-phase arrest, it actually reinforced the latter [27]. Consistent with these studies, our data showed that the formation of gamma-H2AX foci and ROS levels were significantly elevated during RSV induced senescence in breast and lung cancer cells, whereas nucleosides and NAC could each attenuate RSV-induced cellular senescence, which was accompanied by decrease of DNA damage or ROS levels, and downregulation of p21. In addition, both nucleoside and NAC were shown to significantly reduce RSV-mediated upregulations of Rad9 and its target gene p21, which indicated that both DDR- and ROS-dependent pathways are involved in the RSV-mediated upregulation of Rad9. Although the mechanisms of RSV elicited premature senescence leading to increased Rad9 mRNA and protein levels is presently unclear, based on results in this study, we propose that RSV can induce senescence in breast cancer and lung cancer cells by activating DDR and elevating ROS levels. This activation of DDR and elevation of ROS levels can in turn upregulate Rad9 and the subsequently induce its target gene p21, that eventually will result in the induction of cellular senescence. The regulation of Rad9 and the mechanisms of Rad9 upregulation by DDR activation and ROS production are currently being studied.

The EMT is recognized as a key process in cancer progression and metastasis because it not only induces cell migration and invasion, but it can also bypass apoptosis and cellular senescence [31]. RSV has been proven to possess the effect of EMT inhibition in various cancer cell lines including breast and lung cancer cells. Vergara et al demonstrated that RSV inhibits EGF-induced EMT in MCF-7 cells via the inhibition of the activation of EFG-mediated Erk pathway [32]. Tang et al reported that RSV could inhibit IGF-1-mediated cell invasion and migration of MDA-MB-435 via suppression of the PI-3k/Akt pathway [33]. Wang et al also reported that RSV inhibits TGF-β1 induced EMT and suppresses cancer invasion and metastasis by reducing the protein levels of EMT transcription factors Snail and Slug in A549 cells [34]. In addition, Yu et al demonstrated that RSV could effectively inhibit the mesenchymal markers of fibronectin, N-cadherin, and vimentin; and induce the MET by downregulating EMT transcription factor FOXC2 through the regulation of miRNA-520h-mediated signal cascade in CL1-5 and A549 lung cancer cells [35]. Consistent with these studies, our data suggested that RSV can inhibit the invasive and migration abilities of A549 cells, the basal and TPA-induced migration and invasion of MCF-7 cells, as well as the EMT-related gene expressions in both cell lines. We also demonstrated that the knockdown of endogenous Rad9 can significantly reduce RSV-mediated inhibition of migration and invasion abilities of cancer cells, increase epithelial markers and decrease mesenchymal markers in RSV-treated cells. These results, together with the aforementioned data demonstrated the upregulation of Rad9 is involved in RSV-induced cellular senescence, suggested that the upregulation of Rad9 is important for RSV-induced cellular senescence, while simultaneously suppress cellular migration and invasion as well as inhibit EMT in breast and lung cancer cell lines.

Slug is one of the most important EMT transcription factors. Slug expression has been reported to correlate more strongly than Snail expression with E-cadherin suppression in breast carcinomas [36]. Slug has also been reported to play a key role in maintaining the aggravated migration potential of breast cancer stem cells [37] and is more relevant for the generation of breast cancer cells from cancer stem cell phenotype than Snail [38]. In addition, Slug expression was reported to be associated with lung invasion and resistance to target therapy, and a high level of Slug expression mRNA in lung cancer specimens was significantly associated with increased rate of cancer recurrence and decreased survival [39]. The senescence-driving protein p21 acts unquestionably as an EMT inhibitor, and the absence of p21 enables the proliferation of DNA damage cells and promotes tumor progression. In this study, we determined that 12.5–50 μM of RSV treatment could induce the upregulation of Rad9, which is triggered by DDR activation and ROS production, and can lead to the upregulation of p21with concomitant downregulation of Slug to suppressed the tumorigenicities of breast and lung cancer cell lines. Furthermore, the knockdown of Rad9 was shown to significantly reduce RSV-mediated—upregulation of p21 and downregulation of Slug, resulting in the diminishing of the anti-tumor effects of RSV. Our findings presented here suggested that DDR- and ROS-mediated upregulation of Rad9 signaling can play a key role in the antitumorigenic effects of RSV when breast and lung cell lines treated with this dietary phytochemical at 12.5–50 μM. It has been reported that the antitumorigenic effects of RSV are mediated by the upregulating of the cyclic AMP response element (CRE)-binding proteins (CREB)- and activating transcription factor 2 (ATF2)-controlled target genes when HEK293 cells and HepG2 hepatoma cells treated with RSV at 20 μM [40].

It has been documented that therapy induced cellular senescence is correlated with better clinical outcome than therapy induced apoptosis [41]. In agreement with this observation, it was reported that the induction of senescence correlates with treatment outcome in previous animal studies [42]. In this study, we determined that 12.5–50 μM of RSV treatment induced the upregulation of Rad9, which not only it can induce cellular senescence, but also suppress cellular migration and invasion to inhibit EMT in breast and lung cancer cell lines. The expression of Rad9 expression is frequently downregulated in breast and lung cancers, and the loss of Rad9 was associated with acquisition of an invasive phenotype in breast and lung cancer cells. Since using 12.5–50 μM of RSV is clinically achievable based on aforementioned *in vivo* studies [43–49], the RSV induced upregulation of Rad9 in human breast and lung cancer cells could represent a potential novel therapy for breast and lung cancers.

## Supporting information

S1 Fig Full-length images of the western blots illustrated in Fig 2B. (PDF)

S2 Fig Full-length images of the western blots illustrated in Fig 3B. (PDF)

S3 Fig Full-length images of the western blots illustrated in Fig 4B. (PDF)

S4 Fig Full-length images of the western blots illustrated in Fig 5B. (PDF)

S5 Fig Full-length images of the western blots illustrated in Fig 7A and 7B. (PDF)

S6 Fig Full-length images of the western blots illustrated in Fig 8A, 8B, 8C and 8D. (PDF)

S1 Table The values used to build graphs in (A) Fig 1, (B) Fig 2A and 2C, (C) Fig 3A, (D) Fig 4A, (E) Fig 5A, (F) Fig 6B. (PDF)
