# Supplementary material for: Resveratrol induced premature senescence and inhibited epithelial-mesenchymal transition of cancer cells via induction of tumor suppressor Rad9
Source: PLoS One. 2019 Jul 16;14(7):e0219317. doi: 10.1371/journal.pone.0219317 (PMC6634400; doi:10.1371/journal.pone.0219317)
Supplement: S1 Fig — (PDF) [file pone.0219317.s001.pdf]

Supplementary Figures

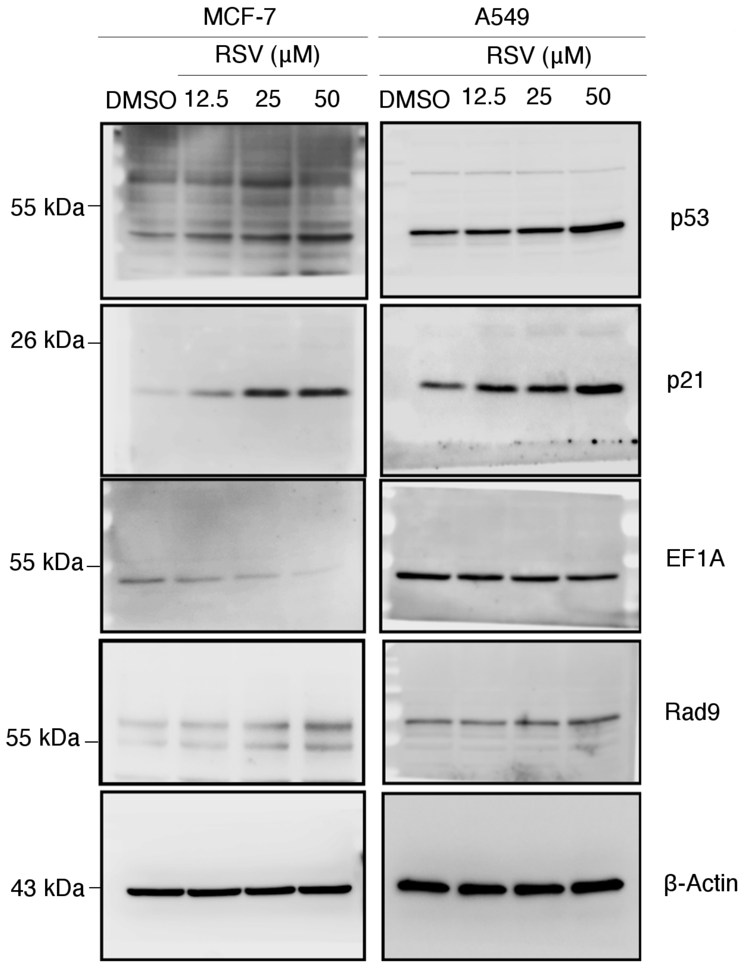

Supplementary Fig S1. Full-length images of the western blots illustrated in Figure 2B.
