# Supplementary figures and images for: Resveratrol induced premature senescence and inhibited epithelial-mesenchymal transition of cancer cells via induction of tumor suppressor Rad9
Source: PLoS One. 2019 Jul 16;14(7):e0219317. doi: 10.1371/journal.pone.0219317 (PMC6634400; doi:10.1371/journal.pone.0219317)

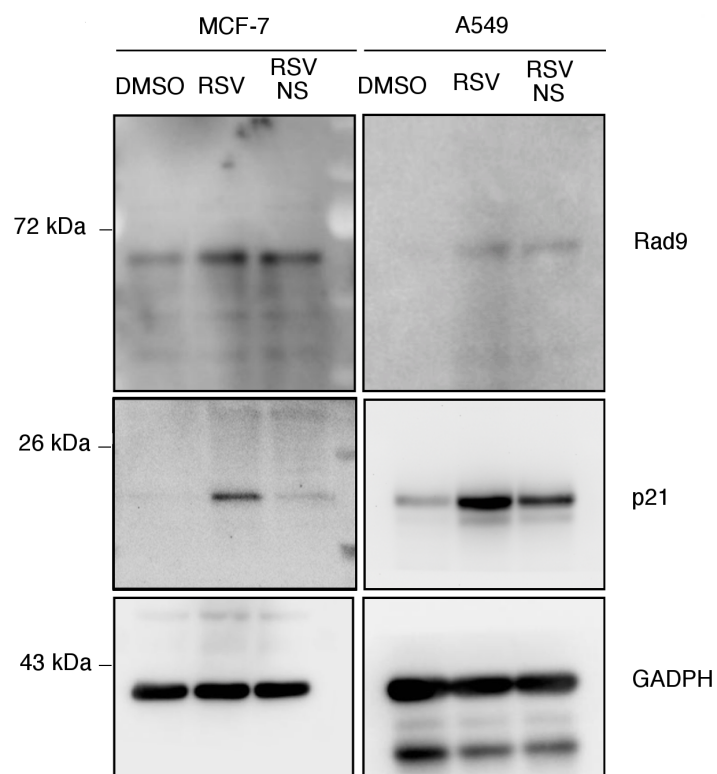

**Supplementary Fig S2.** Full-length images of the western blots illustrated in Figure 3B.

Supplement: S2 Fig — (PDF) [file pone.0219317.s002.pdf]

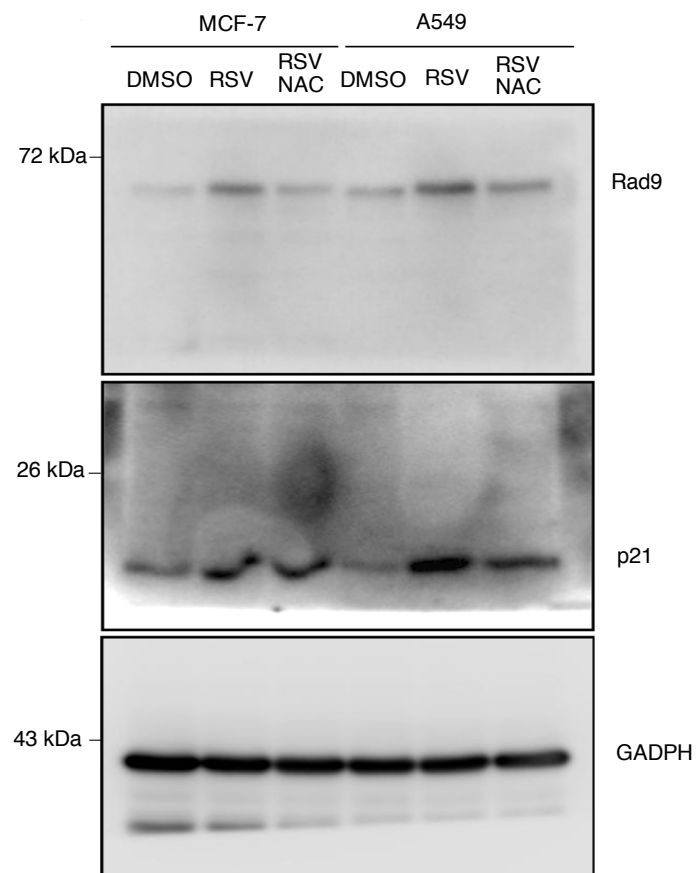

**Supplementary Fig S3.** Full-length images of the western blots illustrated in Figure 4B.

Supplement: S3 Fig — (PDF) [file pone.0219317.s003.pdf]

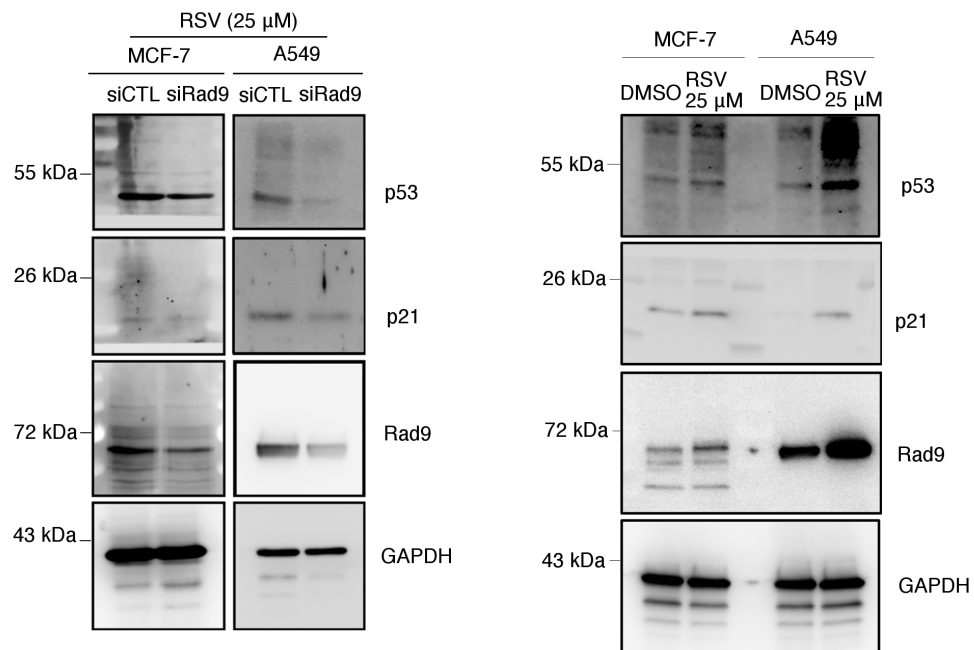

**Supplementary Fig S4.** Full-length images of the western blots illustrated in Figure 5B.

Supplement: S4 Fig — (PDF) [file pone.0219317.s004.pdf]

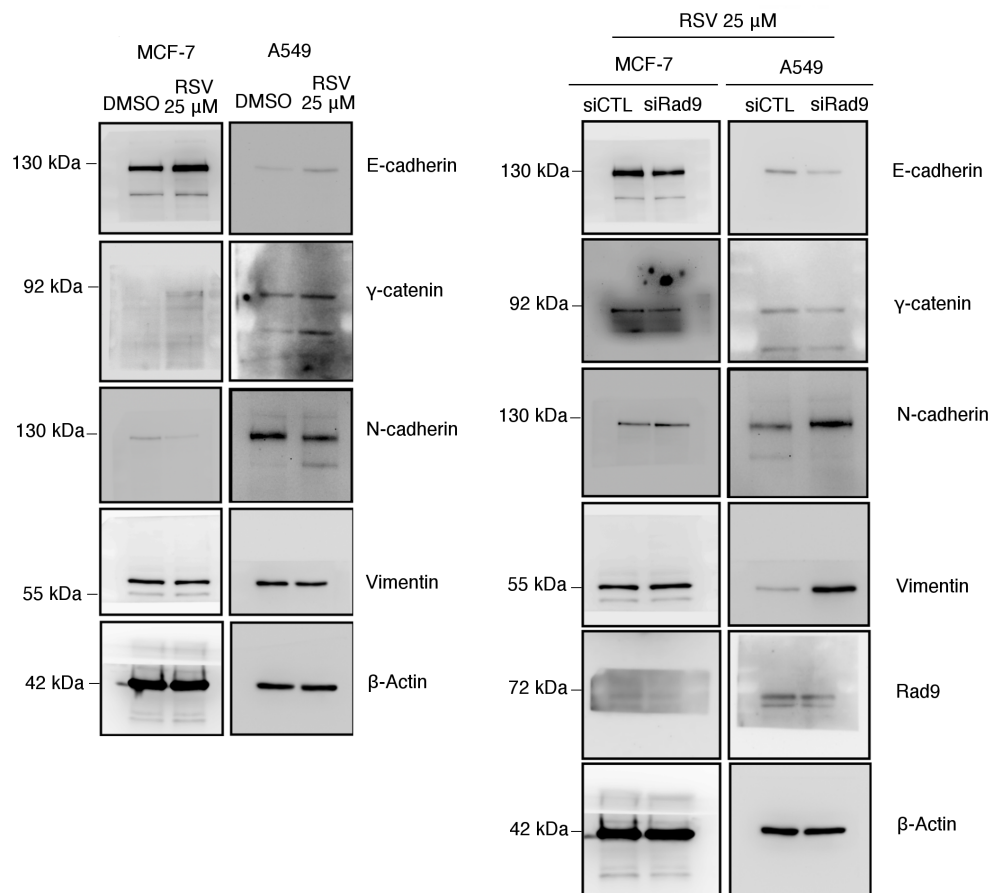

**Supplementary Fig S5.** Full-length images of the western blots illustrated in Figure 7A and B.

Supplement: S5 Fig — (PDF) [file pone.0219317.s005.pdf]

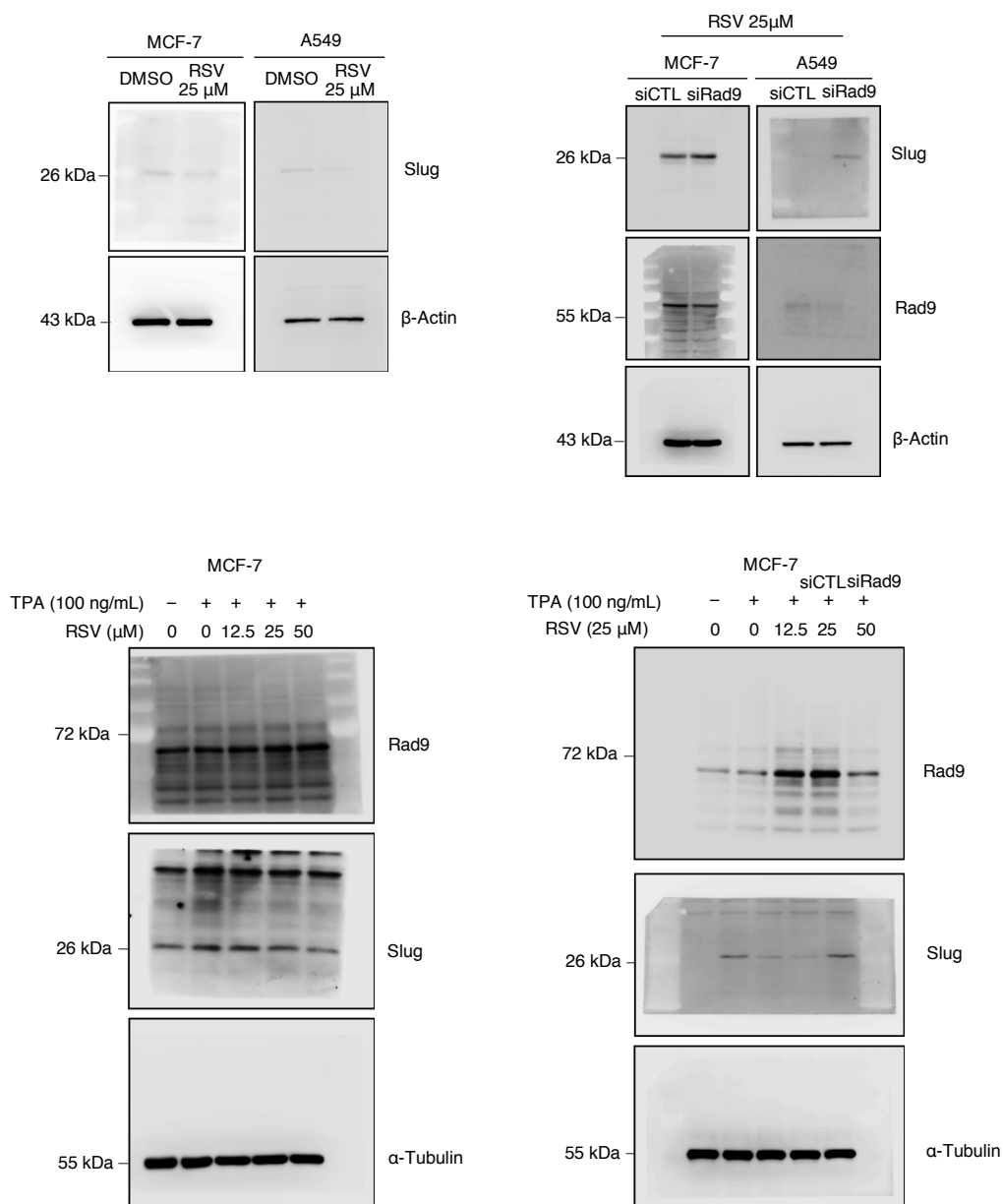

**Supplementary Fig S6.** Full-length images of the western blots illustrated in Figure 8A, B, C and D.

Supplement: S6 Fig — (PDF) [file pone.0219317.s006.pdf]
