# Supplementary material for: Resveratrol induced premature senescence and inhibited epithelial-mesenchymal transition of cancer cells via induction of tumor suppressor Rad9
Source: PLoS One. 2019 Jul 16;14(7):e0219317. doi: 10.1371/journal.pone.0219317 (PMC6634400; doi:10.1371/journal.pone.0219317)
Supplement: S1 Table — (PDF) [file pone.0219317.s007.pdf]

Supplementary Table 1. The values used to build graphs in (A) Figure 1, (B) Figure 2A and 2C, (C) Figure 3A, (D) Figure 4A, (E) Figure 5A, (F) Figure 6B.

A.

	control		RSV 12.5 μM		RSV 25 μM		RSV 50 μM	
cell	MCF-7	A549	MCF-7	A540	MCF-7	A549	MCF-7	A549
cell number	100	100	91	91	77	21	45	2
	105	105	98	89	68	30	39	4
	98	104	101	85	73	28	41	5
mean	101	103	96.67	88.33	72.67	26.33	41.67	3.67
S.D.	2.944	2.16	4.19	2.494	3.682	3.859	2.494	1.25

B.

	DMSO		RSV 25 μM	
cell	MCF-7	A549	MCF-7	A549
positive cell (%)	0.64	1.00	24.00	41.67
	0.78	1.04	40.00	50.00
	0.47	0.95	33.33	28.57
mean	0.63	0.99	32.44	40.08
S.D.	0.13	0.04	6.56	8.82

	DMSO		RSV 25 μM	
cell	MCF-7	A549	MCF-7	A549
relative mRNA level	1.4	1.2	3.78	2.37
	1.3	1.5	4.1	2.55
	1.1	1.2	3.5	2.8
mean	1.27	1.30	3.79	2.57
S.D.	0.12	0.14	0.25	0.18

C.

	DMSO	25 μM RSV	50 nM NS	25μM RSV + 50 nM NS	mean	S.D.
MCF-7 positive cell (%)	0	33	10	10	13.25	12.11
	0	50	10	20	20.00	18.71
	10	50	20	25	26.25	14.74
	10	33	0	25	17.00	12.83
	0	50	0	20	17.50	20.46
A549 positive cell (%)	10	50	0	20	20.00	18.71
	0	33	10	33	19.00	14.44
	0	50	20	25	23.75	17.81
	10	25	10	20	16.25	6.50
	10	33	10	20	18.25	9.44

D.

cell	MCF-7			A549		
condition	DMSO	NAC	NAC + RSV	DMSO	NAC	NAC + RSV
DCF positive cell (%)	12.37	90.48	61.11	3.7	38.98	2.13
	0	85.71	10.43	1.16	56.32	9.62
	0	86.21	21.43	0	56.25	18.97
	0	54.55	13.89	0	63.16	23.86
	0	95.56	11.69	0	60.98	19.15
	0	56.47	16.13	0	53.28	10.2
	0	74.39	30.77	0	54.1	8.06
	0	71.88	58.06	0	55.3	12.1
	0	85.71	21.43	0	53.8	13.5
mean	1.37	77.88	27.22	0.54	54.69	13.07
S.D.	3.89	13.80	18.27	1.17	6.38	6.27

E.

cell	MCF-7		A549	
condition	siCTL	siRad9	siCTL	siRad9
positive cell (%)	30.769	13.636	50	11.905
	35.294	12.121	55.56	10
	46.154	18.75	55.56	10.256
mean	37.41	14.84	53.70	10.72
S.D.	6.46	2.84	2.62	0.84

F.

cell	MCF-7				A549			
condition	DMSO	RSV 25 μM	siCTL	siRad9	DMSO	RSV 25 μM	siCTL	siRad9
cell number	77	34	44	67	110	45	37	88
	82	31	59	74	105	51	21	65
	72	47	50	81	97	47	56	105
mean	77.00	37.33	51.00	74.00	104.00	47.67	38.00	86.00
S.D.	4.08	6.94	6.16	5.72	5.35	2.49	14.31	16.39
